# The Burden of BK Polyomavirus in Pediatric Renal Transplantation: A Belgian Experience

**DOI:** 10.3390/biomedicines14020429

**Published:** 2026-02-13

**Authors:** Pauline Guillaume-Gentil, Benedetta Chiodini, Brigitte Adams, Jean Herman, Maria Van Dyck, Khalid Ismaili

**Affiliations:** 1 Department of Pediatric Nephrology, Hôpital Universitaire de Bruxelles-HUDERF (HUB-HUDERF), Université Libre de Bruxelles (ULB), 1020 Brussels, Belgium; p.guillaumegentil@hubruxelles.be (P.G.-G.); khalid.ismaili@hubruxelles.be (K.I.); 2 Department of Pediatric Nephrology, University Hospitals Leuven (UHL), 3000 Leuven, Belgium

**Keywords:** BK polyomavirus, kidney transplantation, children

## Abstract

**Background/Objectives**: To evaluate the outcome of developing BKPyV-DNAemia and presumptive BKPyV-nephropathy (BKPyV-DNAemia ≥ 10^4^ copies/mL for more than 2 weeks) within the first 2 years post-transplant in a Belgian population of renal transplanted children. **Methods**: All children transplanted between 1 January 2010 and 31 December 2022 at Queen Fabiola Children’s University Hospital, Brussels (HUDERF) and at University Hospitals Leuven (UHL) were included in this retrospective study and 86 were followed for at least 2 years post-transplantation. **Results**: Within the first 2 years, 11/86 (13%) patients developed BKPyV-DNAemia ≥ 10^4^ copies/mL (82% within the first 6 months). Among the 11 patients, 7 underwent a biopsy, of whom 4 were confirmed to have biopsy-proven BKPyV-nephropathy. Of those 11 patients, 4 (36%) developed an acute cellular rejection following immunosuppression reduction. The median eGFR at 2 years post-transplantation was 69 mL/min/1.73 m^2^ (IQR: 59–79) in the seven patients with presumptive BKPyV-nephropathy and 40 mL/min/1.73 m^2^ (IQR: 39–41) in the four with biopsy-proven BKPyV-nephropathy. At last follow-up visit, the median eGFR was 65 mL/min/1.73 m^2^ (IQR: 59–71) in the children with presumptive BKPyV-nephropathy, and 28 mL/min/1.73 m^2^ (IQR: 20–34) in the patients with biopsy-proven BKPyV-nephropathy. No risk factors for developing BKPyV-DNAemia were identified. **Conclusions**: Our study confirms that while BKPyV-DNAemia monitoring is essential in pediatric kidney transplant recipients, decisions based solely on viral load risk overtreatment and immunological complications. A personalized approach integrating viral, clinical, and immunological markers is urgently needed to balance infection control with graft preservation.

## 1. Introduction

BK polyomavirus (BKPyV) is a human double-stranded DNA virus that was first identified in 1971 in the urine of a renal transplant recipient [1]. Primary BKPyV infection typically occurs early in life and is mainly transmitted through horizontal routes, including direct contact, aerosols, and the fecal–oral pathway [2]. Most likely, BKPyV initially replicates in the respiratory tract and subsequently disseminates hematogenously to other organs, particularly renal tubular epithelial and urothelial cells [3]. Approximately 80% of the general population exhibit detectable BKPyV antibodies, most of which appear before the age of 10 years and persist throughout life [4].

BKPyV infection is asymptomatic in immunocompetent hosts, and it remains latent in the urinary tract. In renal transplant recipients, the immunosuppressive treatment disrupts the equilibrium between virus replication and immune response, resulting in potential uncontrolled BKPyV replication [5]. Regarding the source of BKPyV, a graft-derived reactivation has been repeatedly suggested. However, a viral reactivation within the recipient’s urinary tract can also occur, and the two sources may not be mutually exclusive, both in adults as in children [6,7,8,9].

BKPyV replication starts in the distal tubular epithelial cells, leading to necrosis and the initiation of local damage and inflammation. The spread of the virus in the adjacent environment results in BKPyV-DNAuria, which is the first evidence of BKPyV reactivation and appears in 20% to 60% of patients [10]. After this initial stage, disruption of the tubular basement membrane can occur, leading to BKPyV-DNAemia in 10 to 20% of kidney transplant recipients. BKPyV-nephropathy, which is caused by the recruitment of inflammatory cells in the tubule interstitial space and viral spreading to proximal cells, occurs in 1–10% of renal allograft recipients. It appears mainly within the first 2 years post-transplantation and causes graft loss in about half of these patients [3,4,5].

Regarding plasma BKPyV-DNA load, high-level (more than 10^4^ copies/mL) and prolonged (more than 2 weeks) BKPyV-DNAemia seems to increase the risk of BKPyV-nephropathy and is considered to be presumptive for the condition [11,12].

At present, reducing immunosuppression when high-level plasma BKPyV-DNA load appears is the only effective treatment to reduce the risk of BKPyV-nephropathy [5]. However, reducing immunosuppression in renal transplant recipients is not without danger, as it increases the risk of graft rejection [13].

In this scenario, we aim to perform a retrospective multi-centric study in a pediatric population of renal transplanted Belgian children to evaluate the outcome of patients developing high BKPyV-DNAuria, BKPyV-DNAemia, and BKPyV-nephropathy within the first 2 years post-transplant. This study will also try to identify risk factors potentially associated with BKPyV-nephropathy in this population.

## 2. Materials and Methods

### 2.1. Study Population

All patients who underwent a renal transplantation between 1 January 2010 and 31 December 2022 at the Queen Fabiola Children’s University Hospital in Brussels (HUDERF) and at the University Hospitals Leuven (UHL) were included in the study and analyzed retrospectively. Only children with insufficient data for evaluation were excluded. All other children were assigned to one of the following groups according to their BKPyV-DNAemia status: negative, <10^4^ copies/mL, or ≥10^4^ copies/mL.

### 2.2. Definitions

Definitions of BKPyV-DNAuria, BKPyV-DNAemia, and BKPyV-nephropathy were based both on adult international guidelines [14,15] and the Pediatric International CERTAIN Registry [16].

A high level of BKPyV-DNAuria was defined as a urine BKPyV-DNA load ≥ 10^7^ copies/mL. A high level of BKPyV-DNAemia was defined as a plasma BKPyV-DNA load ≥ 10^4^ copies/mL. Presumptive BKPyV-nephropathy was defined as a prolonged (more than 2 weeks) high level of BKPyV-DNAemia. Biopsy-proven BKPyV-nephropathy was defined according to the BANFF classification [14,17]: SV40 T antigen staining, nuclear inclusion, cell infiltration, necrotic tubular epithelium, tubular atrophy and interstitial fibrosis.

### 2.3. Methodology

Data from kidney transplant patients were collected retrospectively from hospital admission and consultation reports, as well as from imaging, biological and anatomo-pathological results. Clinical and virological data were collected from each patient’s medical record at the time of transplantation (day 0) and at 6, 12 and 24 months post-transplant. Data were also collected at last follow-up visit.

The Schwartz formula was used to estimate glomerular filtration rate (eGFR) [18].

The study was approved by the local ethics committees of both hospitals (protocol reference number: CEH n. 88/22 for HUDERF and S69978 for UHL).

### 2.4. Immunosuppression Protocols

Patients were treated in the two centers according to very similar predefined immunosuppressive regimens. Basiliximab (Simulect) was administered intravenously in two doses on days 0 and 4 after transplantation. Calcineurin inhibitors (CNIs) were started on day 0 before transplantation. Cyclosporine microemulsion (Neoral^®^) was given at a starting dose of 4–6 mg/kg/day with subsequent adjustment according to therapeutic drug monitoring. Whole-blood trough concentrations of Cyclosporine were targeted between 150 and 300 ng/mL during the first 3 months post-transplantation and at approximately 150 ng/mL thereafter. Tacrolimus (Prograft^®^) was administered at a starting dose of 0.2 mg/kg/day and trough levels targeted in the range of 8–15 ng/mL during the first 3 months, 5–12 ng/mL until the first year, and 5–10 ng/mL thereafter. Most patients received Mycophenolate Mofetil (MMF, CellCept^®^) at doses of 900 to 1200 mg/m^2^/day. This was progressively reduced in patients with MMF-related adverse effects. Methylprednisolone was administered intravenously at 250 mg/m^2^ and 125 mg/m^2^ at day 0 of transplantation and at the first postoperative day. From the second postoperative day, prednisone was administered orally at 2 mg/kg/day during the first month, and was subsequently progressively tapered and eventually withdrawn in patients who had not developed acute rejection, had good graft function, and were considered at low immunological risk.

### 2.5. Screening and Treatment Strategy of BKPyV Infections

Both centers had an identical strategy for screening and treatment of BKPyV infection. BKPyV infection was systematically screened and investigated in all kidney transplant patients.

From 2010 to 2021, the two centers based the screening and the treatment strategy of BKPyV infections on the viral DNA load in the urine by Polymerase Chain Reaction (PCR). BKPyV viral load in urine was tested once a month for the first 6 months, then once every 3 months for 18 months, then once a year. At first positive BKPyV-DNAuria, the analysis was repeated one month later. If BKPyV-DNAuria was then ≥10^7^ copies/mL, BKPyV viral load in plasma was measured and an initial reduction in immunosuppression was started (trough levels of Tacrolimus targeted below 8 ng/mL). If BKPyV-DNAemia was ≥10^4^ copies/mL with either an increase in plasma creatinine or a prolonged (more than 2 weeks) ≥ 10^4^ copies/mL BKPyV-DNAemia, a second reduction in immunosuppression was recommended (50% reduction in MMF dosage). If BKPyV-DNAemia was <10^4^ copies/mL, BKPyV-DNAemia was then monitored monthly. In our laboratories, BKPyV-DNAemia was quantified using a standard PCR assay performed on the AltoStar^®^ Automation System AM16 (Hamburg, Germany).

**Figure 1:**
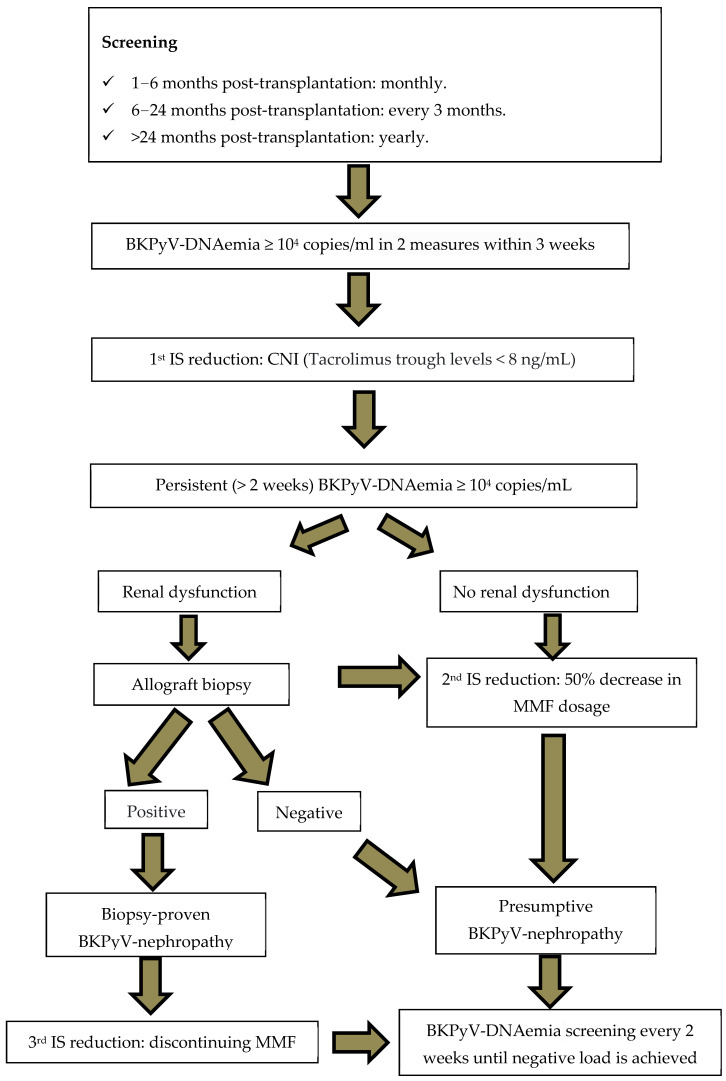
Flow chart of BKPyV screening and management strategy since 2022. Abbreviations: IS = immunosuppression, CNIs = calcineurin inhibitors, MMF = mycophenolate mofetil.

From 2022 onward, the screening strategy changed in both centers and became solely based on plasma viral load (once a month for the first 6 months, then once every 3 months for 18 months, then once a year). The treatment of BKPyV infection and the indication to perform a biopsy to exclude BKPyV-nephropathy remained the same (see Figure 1).

A renal biopsy was performed in case of increase in plasma creatinine to prove the BKPyV-nephropathy and to exclude a graft rejection. If biopsy results were consistent with the diagnosis of BKPyV-nephropathy, a third reduction in the immunosuppressive treatment was recommended (discontinuing the anti-metabolite). If renal biopsy did not prove a BKPyV-nephropathy, a plan for a regular monitoring of BKPyV-DNAemia every 2 weeks (until negative load was achieved) was set up. In refractory cases, a treatment with intravenous immunoglobulins (IV) at a total dose of 2 g/kg or a Cidofovir injection was considered.

### 2.6. Statistical Analysis

Statistics are presented as proportions for categorical variables, means with standard deviations for normally distributed continuous variables, and medians with the interquartile range for continuous variables that did not follow a normal distribution. Differences between study groups are tested using the independent-sample Mann–Whitney U test for non-normally distributed continuous variables, two-tailed Student’s *t*-test for normally distributed continuous variables and Fisher’s exact test for categorical variables. A value of *p* < 0.05 is considered statistically significant [19].

## 3. Results

### 3.1. Characteristics of the Patients

From January 2010 to December 2022, a total of 90 renal transplantations were performed in the two centers. Their clinical characteristics are shown in Table 1.

Four patients dropped out from the analysis: three patients were lost to follow-up and one lost his graft at day 6 after surgery for a renal arterial thrombosis.

A total of 86/90 (96%) patients were followed for at least 2 years after transplantation.

### 3.2. Results in Terms of BKPyV-DNAuria During the 2 Years Post-Transplantation

A total of 45/86 (52%) patients developed a BKPyV-DNAuria within the first 2 years post-transplantation: 14/86 (16%) a BKPyV-DNAuria < 10^7^ copies/mL and 31/86 (36%) had BKPyV-DNAuria ≥ 10^7^ copies/mL. The development of high-level BKPyV-DNAuria appeared within the first 6 months for 21/31 (68%) of them and during the first-year post-transplantation for 28/31 (90%).

Among those 31 children with high-level BKPyV-DNAuria, 12 (39%) never showed BKPyV-DNAemia, 11 (35%) progressed to BKPyV-DNAemia < 10^4^ copies/mL and 8 (26%) developed BKPyV-DNAemia ≥ 10^4^ copies/mL.

### 3.3. Results in Terms of BKPyV-DNAemia During the First 2 Years Post-Transplantation and at Last Follow-Up Visit

**Figure 2:**
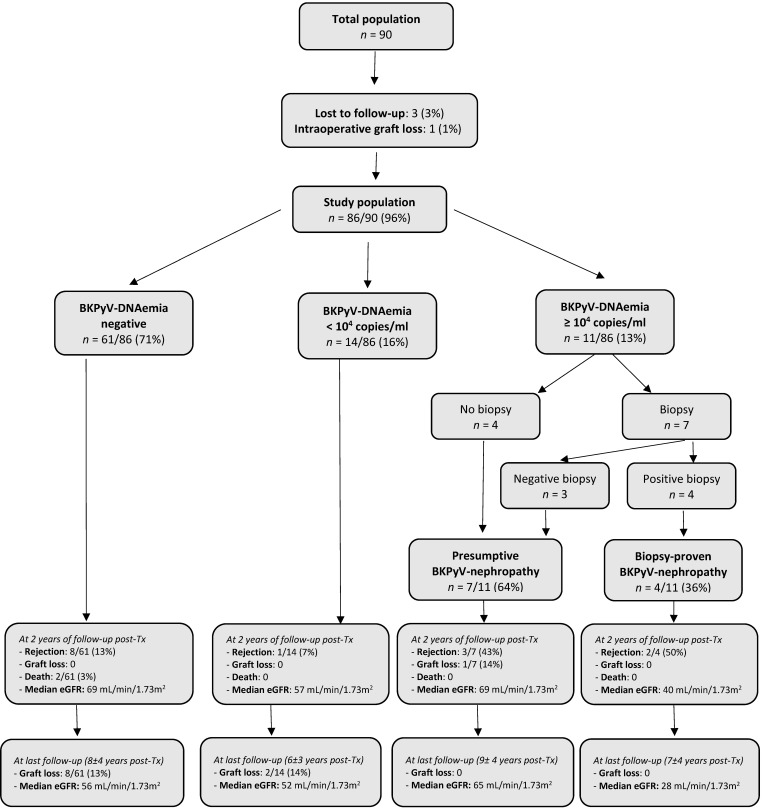
Flow chart of the population at 2 years after transplantation and at last follow-up. Abbreviations: Tx = transplantation, eGFR = estimated glomerular filtration rate.

The flow chart (see Figure 2) shows the clinical and virological evolution of the cohort.

During the first 2 years post-transplantation, BKPyV-DNAemia was not detected in 61/86 (71%) patients. Among them, 8/61 (13%) children had a graft rejection (6 acute cellular, 1 humoral and 1 chronic rejection) at an average time of 10 months post-transplantation. No graft was lost during this period. Two children died: the first from a post-transplant lymphoproliferative disorder at 8 months post-surgery and the second one from an hepatoblastoma at 14 months post-transplantation. The median eGFR at 2-year follow-up was 69 mL/min/1.73 m^2^ (IQR: 54–77).

#### 3.3.1. Children Without BKPyV-DNAemia (*n* = 61)

At last follow-up visit at a mean time of 8 ± 4 years post-transplantation, the median eGFR was 56 mL/min/1.73 m^2^ (IQR: 38–68).

Among this group, 8/61 (13%) lost their graft at a median time of 5.6 (IQR: 4.7–8.0) years after transplantation and went back to dialysis because of chronic rejection and/or CNI toxicity.

#### 3.3.2. Children with Positive BKPyV-DNAemia (*n* = 25)

During the first 2 years post-transplantation, a total of 25/86 (29%) children developed BKPyV-DNAemia: 14 maintained BKPyV-DNAemia < 10^4^ copies/mL and 11 progressed to ≥10^4^ copies/mL.

#### 3.3.3. Children with BKPyV-DNAemia < 10^4^ Copies/mL (*n* = 14)

During the first 2 years post-transplantation, a total of 14/86 (16%) children presented BKPyV-DNAemia < 10^4^ copies/mL at a median time of 4.3 months (IQR: 2–7).

Of those 14 patients, 11 (79%) developed it within the first 6 months post-transplantation, 2 (14%) at 7 and 8 months respectively, and 1 (7%) at 20 months post-transplantation.

Of those 14 patients, 1 had an acute cellular rejection at 10 months post-surgery. None lost their graft or died during this period. The median eGFR at 2-year follow-up was 57 mL/min/1.73 m^2^ (IQR: 46–81).

At last follow-up visit, at a mean time of 7 ± 3 years post-transplantation, the median eGFR was 52 mL/min/1.73 m^2^ (IQR: 34–70).

Among this group, 2/14 (14%) patients lost their grafts at 3 and 5 years post-transplantation and went back to dialysis because of cellular and humoral rejections, respectively.

#### 3.3.4. Children with BKPy-DNAemia ≥ 10^4^ Copies/mL (*n* = 11)

During the first 2 years post-transplantation, 11/86 (13%) children developed BKPyV-DNAemia ≥ 10^4^ copies/mL at a median time of 4.5 months (IQR: 4–9).

Of those 11 patients, 4 (36%) did not undergo a biopsy because their plasma creatinine remained stable, while 7 (64%) underwent a biopsy because of degradation of their renal function. Of the seven children who underwent the biopsy, four showed biopsy-proven BKPyV-nephropathy and three with a negative finding.

Children with a persisting (>2 weeks) BKPyV-DNAemia ≥ 10^4^ copies/mL who either did not undergo the biopsy or showed a negative result were classified as presumptive BKPyV-nephropathy (*n* = 7). Those with a confirming biopsy were classified as having a biopsy-proven BKPyV-nephropathy (*n* = 4).

Of the seven children classified as presumptive BKPyV-nephropathy, five (71%) developed it within the first 6 months post-transplantation, all within the first year. Within this presumptive BKPyV-nephropathy group, three (43%) children developed an acute rejection at a mean time of 11 ± 8 months post-transplantation, and one patient lost his graft at 1.6 years post-transplantation because of a vascular complication during the treating procedure of a renal arterial stenosis. None of them died during the follow-up period. The median eGFR at 2 years post-transplantation was 69 mL/min/1.73 m^2^ (IQR: 59–79).

Of the four patients who progressed towards a biopsy-proven BKPyV-nephropathy, half were diagnosed during the first year and the other half within the second post-transplantation year. Half of the children within this biopsy-proven BKPyV-nephropathy group also developed an acute rejection at a mean time of 15 ± 12 months post-transplantation. None of them died during the follow-up period. The median eGFR at 2 years post-transplantation was 40 mL/min/1.73 m^2^ (IQR: 39–41), significantly lower compared to the presumptive BKPyV-nephropathy group (*p*-value of <0.01).

At last follow-up visit, the median eGFR was 65 mL/min/1.73 m^2^ (IQR: 59–71) for the presumptive BKPyV-nephropathy group and 28 mL/min/1.73 m^2^ (IQR: 20–34) for the biopsy-proven BKPyV-nephropathy group (*p*-value of <0.001). None of the patients lost their graft after 2 years post-transplantation.

### 3.4. Management of Patients Showing BKPyV-DNAemia ≥ 10^4^ Copies/mL

The characteristics of the 11 patients with BKPyV-DNAemia ≥ 10^4^ copies/mL are resumed in Table 2. All children in this group showed a reduction in their immunosuppression. Additional treatment was provided for the four children with biopsy-proven BKPyV-nephropathy: three received intravenous immunoglobulins and one received Cidofovir.

In total, 4/11 (36%) developed acute cellular rejection after reducing the immunosuppression.

### 3.5. Risk Factors of Developing a BKPyV-DNAemia ≥ 10^4^ Copies/mL

The following variables have been evaluated as potential risk factors for developing a BKPyV-DNAemia ≥ 10^4^ copies/mL: male sex, primary kidney disease, age of recipient and donor, donor and recipient CMV and EBV immunity status before transplant, cold and warm ischemic time, and double J stent placement (see Table 3).

None of these variables were significant risk factors for developing BKPyV-DNAemia ≥ 10^4^ copies/mL in this population of renal transplanted children.

## 4. Discussion

The purpose of our study was to evaluate the prevalence and outcome of developing high BKPyV-DNAuria, BKPyV-DNAemia and BKPyV-nephropathy within the first 2 years post-transplant in a Belgian population of renal transplanted children.

Our findings align closely with data from major registries and international guidelines. During the first 2 years post-transplantation, we observed rates of BKPyV-DNAuria ≥ 10^7^ copies/mL, BKPyV-DNAemia ≥ 10^4^ copies/mL, and biopsy-proven BKPyV-nephropathy of 36%, 13%, and 5%, respectively—comparable to those reported in the NAPRTCS [20] and the European CERTAIN registry [16]. Viral reactivation mostly appeared in the first 6–12 months post-transplant, supporting current guidelines for intensified early plasma BKPyV-DNA monitoring [5,14,15].

Consistent with the recent Transplantation Society International BK Polyomavirus Consensus Group TTS [15], our results indicate that urine BKPyV-DNA lacks predictive specificity. Three-fourths of children with high-level BKPyV-DNAuria never developed BKPyV-DNAemia or graft dysfunction, reinforcing updated recommendations to deprioritize urine monitoring in centers where reliable plasma assays exist. Shifting to plasma-only surveillance in 2022 reduced unnecessary testing and cost without compromising patient outcomes.

Among the 11 patients with a sustained BKPyV-DNAemia ≥ 10^4^ copies/mL, only 7 underwent a biopsy (all prompted by rising creatinine) and 4 of them were eventually histologically confirmed. This selective biopsy approach may have missed subclinical BKPyV-nephropathy or led to overtreatment based on BKPyV-DNAemia alone. Within the first 2 years post-transplantation, a third of these patients with a BKPyV-DNAemia ≥ 10^4^ copies/mL developed acute cellular rejection following immunosuppression reduction. This result is consistent with other studies [13] linking BKPyV-DNAemia-guided immunosuppression reduction to increased risks of rejection, donor-specific antibodies, and graft dysfunction. These observations, in line with Hirsch et al. [14] and the TTS guidelines [15], underscore a key limitation: viral load-based presumptive BKPyV-nephropathy lacks the precision required for safe immunosuppression adjustments.

Because of the poor outcome of the biopsy-proven BKPyV-nephropathy and because of the incidence of rejection post-immunosuppression reduction, early markers are crucial for identifying kidney transplant recipients at risk of progression from presumptive to definitive BKPyV-nephropathy. Their timely detection is essential to guiding clinical management and preventing irreversible graft damage.

Efforts are increasingly focusing on integrating predictive biomarkers into clinical care.

Recent research has highlighted the importance of BKPyV-specific cell-mediated immunity in controlling viral replication after kidney transplantation. Studies in both adults and children have shown that increased levels of BKPyV-specific T cells are linked to a higher chance of viral clearance [21,22,23]. Monitoring this immune response has been proposed as a prognostic tool for identifying patients at risk of BKPyV-nephropathy [5].

A recent study in a German pediatric transplant cohort found that low or absent BKPyV-specific CD4 and CD8 T cells were associated with persistent BKPyV-DNAemia and severe BKPyV-nephropathy, while adequate levels were linked to mild, self-limiting infections [24].

Future pediatric-specific prospective trials should include BKPyV-specific immune profiling and early injury markers to guide more nuanced management.

Combining these biomarkers with BKPyV-DNAemia could refine decision-making, improving outcomes and reducing graft dysfunction in pediatric transplant recipients.

Currently, routine use of this immune monitoring is still limited by cost, accessibility, and technical challenges.

Another approach to predict the course of BKPyV infection could be to assess the humoral immune response. Several studies have shown that high titers of BKPyV-specific antibodies directed against the donor strains (mostly serotypes I and IV) prior to transplantation may have a protective effect and may therefore be associated with a reduced risk of post-transplant BKPyV infection [25,26,27]. However, this finding is still controversial as other studies have suggested a limited role of the humoral immune response in controlling BKPyV replication and virulence [10,28,29].

Different studies demonstrated an association between an increased risk to develop BKPyV-DNAemia and BKPyV-nephropathy and risk factors, such as younger age at kidney transplantation, male sex, obstructive uropathy, intensity of immunosuppression therapy, Tacrolimus and MMF association, ureteral stent placement at transplantation, prolonged cold ischemia, and CMV seronegative recipient before transplantation [10,16,23,30,31,32,33]. However, none of these risk factors achieved significance in our population, most probably because of the small sample size.

Our study presents clear limitations. The retrospective design and the limited number of biopsy-confirmed BKPyV-nephropathy cases reduces statistical power.

Biopsies were performed selectively—mainly in patients with renal dysfunction—potentially underdiagnosing subclinical cases.

Although both centers followed similar protocols, subtle variations in clinical decision-making may have influenced outcomes.

However, our study presents also substantial strengths.

Unlike registry studies (e.g., NAPRTCS, CERTAIN) [16,20] that included heterogeneous populations, varied BKPyV definitions, and inconsistent immunosuppressive practices, our study features a homogeneous pediatric cohort with well-defined criteria, centralized protocols, and synchronized management across two centers. Therefore, our comparable rates of presumptive and biopsy-proven BKPyV-nephropathy are consistent with and support the heterogeneous findings reported in registry studies.

Regular scheduling at predefined time points enhance the internal validity of our findings and reduce clinical bias.

## 5. Conclusions

Our study confirms that while BKPyV-DNAemia monitoring is essential in pediatric kidney transplant recipients, decisions based solely on viral load risk overtreatment and immunological complications. A personalized approach that integrates viral, clinical, and immunological markers is urgently needed to balance infection control with graft preservation.

## Figures and Tables

**Table 1:** Clinical characteristics of the pediatric renal transplant recipients (*n* = 90) between January 2010 and December 2022.

Characteristics	Total Population *n* = 90	HUDERF Population *n* = 55 (61%)	UHL Population *n* = 35 (39%)	*p*-Value
Male sex, *n* (%)	44 (49%)	29 (53%)	15 (43%)	0.36
Primary kidney disease, *n* (%)				
▪ Congenital anomalies of the kidney and urinary tract	28 (31%)	22 (40%)	6 (17%)	0.02
▪ Nephrotic syndrome of genetic origin	18 (20%)	11 (20%)	7 (20%)	1
▪ Ciliopathy	20 (22%)	9 (16%)	11 (31%)	0.09
▪ Other *	24 (27%)	13 (24%)	11 (31%)	0.4
Age of recipient (years), median (IQR)	11 (5–14)	11 (5–14)	11 (5–14)	0.98
Age of donor (years), median (IQR)	31 (20–39)	33 (21–40)	25 (20–34)	0.15
Living donor, *n* (%)	29 (32%)	17 (31%)	12 (34%)	0.74
Type of renal replacement therapy before transplant, *n* (%)				
▪ Hemodialysis	50 (56%)	28 (51%)	22 (63%)	0.13
▪ Peritoneal dialysis	17 (19%)	13 (24%)	4 (11%)	
▪ Peritoneal dialysis + hemodialysis	8 (9%)	7 (13%)	1 (3%)	
▪ Pre-emptive transplant	15 (17%)	7 (13%)	8 (23%)	
Previous transplantation, *n* (%)	7 (8%)	3 (5%)	4 (11%)	0.2
History of kidney transplantation rejection, *n* (%)	1 (1%)	1 (2%)	0 (0%)	1
Immunity status before transplant, *n* (%)				
▪ CMV: R−/D+	21 (23%)	14 (25%)	7 (20%)	0.55
▪ EBV: R−	39 (44%)	23 (42%)	16 (47%)	0.63
Warm ischemia time (minutes),				
median [IQR]	36 (30–50)	40 (30–54)	33 (30–40)	0.08
Cold ischemia time (minutes),				
median [IQR]	698 (205–900)	674 (236–899)	720 (55–901)	0.58
Immunosuppressive treatment, *n* (%)				0.51
▪ Basiliximab + MMF + Tacrolimus + Prednisone	76 (84%)	48 (87%)	28 (80%)	
▪ Basiliximab + Azathioprine + Tacrolimus + Prednisone	4 (5%)	1 (2%)	3 (8%)	
▪ Basiliximab + MMF + Ciclosporine + Prednisone	3 (3%)	2 (4%)	1 (3%)	
▪ Thymoglobuline + MMF + Tacrolimus + Prednisone	7 (8%)	4 (7%)	3 (8%)	
Double J stent, *n* (%)	65 (72%)	30 (55%)	35 (100%)	<0.001
Length of hospitalization (days),				
median (IQR)	18 (15–24)	18 (14–23)	21 (15–25)	0.14

Abbreviations: IQR = interquartile range, CMV = Cytomegalovirus, EBV = Epstein–Barr virus, R− = negative recipient, D+ = positive donor, MMF = mycophenolate mofetil. Legend: Other *: steroid-resistant nephrotic syndrome (*n* = 5), primary hyperoxaluria (*n* = 3), methylmalonic acid (*n* = 3), mitochondrial disease (*n* = 2), IgA nephropathy (*n* = 2), hemolytic uremic syndrome (*n* = 2), renal amyloidosis (*n* = 1), nephrangiosclerosis (*n* = 1), FHHNC syndrome (*n* = 1), tubular necrosis from neonatal asphyxia (*n* = 1), ANCA glomerulopathy (*n* = 1), unknown (*n* = 2).

**Table 2:** Clinical characteristics of children with BKPyV-DNAemia ≥ 10^4^ copies/mL (*n* = 11) within the first 2 years post-transplantation.

Patient Number	First BKPyV-DNAemia (Months)	Biopsy-Proven BKPyV-Nephropathy (Months)	Rejection Post-IS Reduction (Months)	Intravenous Immunoglobulins (g/kg)	eGFR at 2 Years Post-Transplantation (mL/min/1.73 m^2^)	Last Follow-Up Post-Transplantation (Years)	eGFR at Last Follow-Up (mL/min/1.73 m^2^)	Graft Failure (Years)
1	2	NA	NO	NO	105	11.2	68	NO
2	10.5	NA	NO	NO	60	9	72	NO
3	1.5	NA	NO	NO	88	8.7	83	NO
4	8.5	NA	NO	NO	67	14.9	58	NO
5	1	NO	NO	NO	<15	1.6	<15	YES (1.6) ^a^
6	1	NO	YES (9)	NO	60	8	61	NO
7	5.5	NO	YES (19)	NO	59	7.4	55	NO
8	3	YES (3.8)	YES (6.5)	NO	42	12.7	21	NO
9	1	YES (12)	YES (29)	YES (0.8)	55	6.3	15	NO
10	6	YES (24)	NO	YES (0.8)	60	5.5	35	NO
11	3	YES (24)	NO	YES (1.5)	56	4	34	NO

Legend: NA: Not Applicable as no biopsy was performed. **^a^**: Graft failure due to a vascular complication during the treatment procedure of a renal arterial stenosis.

**Table 3:** Risk factors for developing a BKPyV-DNAemia ≥ 10^4^ copies/mL (*n* = 11) in the pediatric renal transplant population (*n* = 86) within the first 2 years post-transplantation.

Risk Factors	BKPyV-DNAemia < 10^4^ Copies/mL and/or Negative *n* = 75 (87%)	BKPyV-DNAemia ≥ 10^4^ Copies/mL *n* = 11 (13%)	*p*-Value
Male sex, *n* (%)	37 (49%)	6 (55%)	0.8
Primary kidney disease, *n* (%)			
▪ Congenital anomalies of the kidney and urinary tract	22 (29%)	4 (36%)	0.7
▪ Nephrotic syndrome of genetic origin	14 (19%)	4 (36%)	0.2
▪ Ciliopathy	17 (23%)	1 (9%)	0.3
▪ Other *	22 (29%)	2 (18%)	0.4
Age of recipient (years), mean (± SD)	10 (5)	8 (5)	0.1
Age of donor (years), mean (± SD)	29 (12)	33 (14)	0.4
Immunity status before transplant, *n* (%)			
▪ CMV: R−/D+	19 (25%)	0 (0%)	0.1
▪ EBV: R+	39 (52%)	4 (36%)	0.3
Warm ischemia time (minutes), median (IQR)	39 (30–51)	34 (32–40)	0.9
Cold ischemia time (minutes), median (IQR)	681 (203–895)	896 (576–962)	0.3
Double J stent, *n* (%)	52 (69%)	10 (91%)	0.1

**Abbreviations:** IQR = interquartile range, CMV = Cytomegalovirus, EBV = Epstein–Barr virus, R−/+ = negative/positive recipient, D+ = positive donor. Legend: Other *: steroid-resistant nephrotic syndrome (*n* = 5), primary hyperoxaluria (*n* = 3), methylmalonic acid (*n* = 3), mitochondrial disease (*n* = 2), IgA nephropathy (*n* = 2), hemolytic uremic syndrome (*n* = 2), renal amyloidosis (*n* = 1), nephroangiosclerosis (*n* = 1), FHHNC syndrome (*n* = 1), tubular necrosis from neonatal asphyxia (*n* = 1), ANCA glomerulopathy (*n* = 1), unknown (*n* = 2).

## Data Availability

The original contributions presented in this study are included in the article. Further inquiries can be directed to the corresponding author(s).

## References

[1] Gardner S., Field A.M., Coleman D.V., Hulme B. (1971). New human papovavirus (B.K.) isolated from urine after renal transplantation. Lancet.

[2] Goudsmit J., Wertheim-van Dillen P., van Strien A., van der Noordaa J. (1982). The role of BK virus in acute respiratory tract disease and the presence of BKV DNA in tonsils. J. Med. Virol..

[3] Boldorini R., Veggiani C., Barco D., Monga G. (2005). Kidney and urinary tract polyomavirus infection and distribution: Molecular biology investigation of 10 consecutive autopsies. Arch. Pathol. Lab. Med..

[4] Stolt A., Sasnauskas K., Koskela P., Lehtinen M., Dillner J. (2003). Sero-epidemiology of the human polyomaviruses. J. Gen. Virol..

[5] Chiodini B., Guillaume-Gentil P., Vanhomwegen C., Hennaut E., Lolin K., Tram N., Le Moine A., Ismaili K. (2024). BK Polyomavirus in Pediatric Renal Transplantation—What We Know and What We Do Not. Biomedicines.

[6] Schmitt C., Raggub L., Linnenweber-Held S., Adams O., Schwarz A., Heim A. (2014). Donor origin of BKV replication after kidney transplantation. J. Clin. Virol..

[7] Gras J., Nere M.L., Peraldi M.N., Bonnet-Madin L., Salmona M., Taupin J.L., Desgrandchamps F., Verine J., Brochot E., Amara A. (2023). BK virus genotypes and humoral response in kidney transplant recipients with BKV-associated nephropathy. Transpl. Infect. Dis..

[8] Mineeva-Sangwo O., Martí-Carreras J., Cleenders E., Kuypers D., Maes P., Andrei G., Naesens M., Snoeck R. (2022). Polyomavirus BK genome comparison shows high genetic diversity in kidney transplant recipients three months after transplantation. Viruses.

[9] Lamarche C., Orio J., Collette S., Senécal L., Hébert M.J., Renoult É., Tibbles L.A., Delisle J.S. (2016). BK polyomavirus and the transplanted kidney: Immunopathology and therapeutic approaches. Transplantation.

[10] Sharma R., Tzetzo S., Patel S., Zachariah M., Sharma S., Melendy T. (2016). BK Virus in Kidney Transplant: Current Concepts, Recent Advances, and Future Directions. Exp. Clin. Transplant..

[11] Cleenders E., Koshy P., Van Loon E., Lagrou K., Beuselinck K., Andrei G., Crespo M., De Vusser K., Kuypers D., Lerut E. (2023). An observational cohort study of histological screening for BK polyomavirus nephropathy following viral replication in plasma. Kidney Int..

[12] Elfadawy N., Flechner S.M., Schold J.D., Srinivas T.R., Poggio E., Fatica R., Avery R., Mossad S.B. (2014). Transient versus Persistent BK Viremia and Long-Term Outcomes after Kidney and Kidney–Pancreas Transplantation. Clin. J. Am. Soc. Nephrol..

[13] Karatas M., Tatar E., Okut G., Yildirim A.M., Kocabas E., Alkan F.T., Simsek C., Dogan S.M., Uslu A. (2024). Efficacy of mTOR Inhibitors and Intravenous Immunoglobulin for Treatment of Polyoma BK Nephropathy in Kidney Transplant Recipients: A Biopsy-Proven Study. Exp. Clin. Transplant..

[14] Hirsch H.H., Randhawa P.S. (2019). AST Infectious Diseases Community of Practice BK polyomavirus in solid organ transplantation—Guidelines from the American Society of Transplantation Infectious Diseases Community of Practice. Clin. Transplant..

[15] Kotton C.N., Kamar N., Wojciechowski D., Eder M., Hopfer H., Randhawa P., Sester M., Comoli P., Tedesco Silva H., Knoll G. (2024). The Second International Consensus Guidelines on the Management of BK Polyomavirus in Kidney Transplantation. Transplantation.

[16] Höcker B., Schneble L., Murer L., Carraro A., Pape L., Kranz B., Oh J., Zirngibl M., Dello Strologo L., Büscher A. (2019). Epidemiology of and Risk Factors for BK Polyomavirus Replication and Nephropathy in Pediatric Renal Transplant Recipients: An International CERTAIN Registry Study. Transplantation.

[17] Nickeleit V., Singh H.K., Randhawa P., Drachenberg C., Bhatnagar R., Bracamonte E., Chang A., Chon W.J., Dadhania D., Davis V.G. (2018). The Banff Working Group Classification of Definitive Polyomavirus Nephropathy: Morphologic Definitions and Clinical Correlations. J. Am. Soc. Nephrol..

[18] Schwartz G.J., Munoz A., Schneider M.F., Mak R.H., Kaskel F., Warady B.A., Furth S.L. (2009). New Equations to Estimate GFR in Children with CKD. J. Am. Soc. Nephrol..

[19] Medistica (2019). Pvalue.io, a Graphic User Interface to the R Statistical Analysis Software for Scientific Medical Publications. https://www.pvalue.io/fr.

[20] Smith J.M., Dharnidharka V.R., Talley L., Martz K., McDonald R.A. (2007). BK Virus Nephropathy in Pediatric Renal Transplant Recipients: An Analysis of the North American Pediatric Renal Trials and Collaborative Studies (NAPRTCS) Registry. Clin. J. Am. Soc. Nephrol..

[21] Ahlenstiel-Grunow T., Pape L. (2020). Diagnostics, treatment, and immune response in BK polyomavirus infection after pediatric kidney transplantation. Pediatr. Nephrol. Berl. West..

[22] Ahlenstiel-Grunow T., Pape L. (2021). Virus-specific T cells in pediatric renal transplantation. Pediatr. Nephrol..

[23] Kant S., Dasgupta A., Bagnasco S., Brennan D.C. (2022). BK Virus Nephropathy in Kidney Transplantation: A State-of-the-Art Review. Viruses.

[24] Ahlenstiel-Grunow T., Sester M., Sester U., Hirsch H.H., Pape L. (2020). BK Polyomavirus-specific T Cells as a Diagnostic and Prognostic Marker for BK Polyomavirus Infections After Pediatric Kidney Transplantation. Transplantation.

[25] Wunderink H.F., Van der Meijden E., Van der Blij-de Brouwe C.S., Mallat M.J., Haasnoot G.W., Van Zwet E.W., Claas E.C., de Fijter J.W., Kroes A.C., Arnold F. (2017). Pretransplantation Donor-Recipient Pair Seroreactivity Against BK Polyomavirus Predicts Viremia and Nephropathy After Kidney Transplantation. Am. J. Transplant..

[26] Solis M., Velay A., Porcher R., Domingo-Calap P., Soulier E., Joly M., Meddeb M., Kack-Kack W., Moulin B., Bahram S. (2018). Neutralizing Antibody-Mediated Response and Risk of BK Virus-Associated Nephropathy. J. Am. Soc. Nephrol..

[27] Dakroub F., Touzé A., Sater F.A., Fiore T., Morel V., Tinez C., Helle F., François C., Choukroun G., Presne C. (2022). Impact of pre-graft serology on risk of BKPyV infection post-renal transplantation. Nephrol. Dial. Transplant..

[28] Chen Y., Trofe J., Gordon J., Du Pasquier R.A., Roy-Chaudhury P., Kuroda M.J., Woodle E.S., Khalili K., Koralnik I.J. (2006). Interplay of cellular and humoral immune responses against BK virus in kidney transplant recipients with polyomavirus nephropathy. J. Virol..

[29] Kaur A., Wilhelm M., Wilk S., Hirsch H.H. (2019). BK polyomavirus-specific antibody and T-cell responses in kidney transplantation: Update. Curr. Opin. Infect. Dis..

[30] Thomas A., Dropulic L.K., Hafizur Rahman M., Geetha D. (2007). Ureteral Stents: A novel risk factor for polyomavirus nephropathy. Transplantation.

[31] Lin F., Zhang Z., Wang C., Liu F., Chen R., Chen J., Fang X., Sun Y., Zhai Y., Xu H. (2024). Risk factors and outcome of BK polyomavirus infection in pediatric kidney transplantation. Pediatr. Nephrol..

[32] Hirsch H.H., Knowles W., Dickenmann M., Passweg J., Klimkait T., Mihatsch M.J., Steiger J. (2002). Prospective Study of Polyomavirus Type BK Replication and Nephropathy in Renal-Transplant Recipients. N. Engl. J. Med..

[33] Gately R., Milanzi E., Lim W., Teixeira-Pinto A., Clayton P., Isbel N., Johnson D.W., Hawley C., Campbell S., Wong G. (2023). Incidence, Risk Factors, and Outcomes of Kidney Transplant Recipients with BK Polyomavirus-Associated Nephropathy. Kidney Int. Rep..

